# National clinical practice guidelines for food allergy and anaphylaxis: an international assessment

**DOI:** 10.1186/s13601-017-0161-z

**Published:** 2017-07-25

**Authors:** Asiyah Sheikh, Zakariya Sheikh, Graham Roberts, Antonella Muraro, Sangeeta Dhami, Aziz Sheikh

**Affiliations:** 1 Research Students, Edinburgh, UK; 2 Clinical and Experimental Sciences and Human Development in Health Academic Unit, University of Southampton Faculty of Medicine, Southampton, UK; 3 Food Allergy Referral Centre Veneto Region Department of Women and Child Health Padua General University Hospital, Padua, Italy; 4 Edinburgh, UK; 5 Asthma UK Centre for Applied Research, The University of Edinburgh, Edinburgh, UK

## Abstract

**Background:**

Clinical practice guidelines are important tools to promote evidence-based clinical care, but not all countries have the capacity or infrastructure to develop these in-house. The European Academy of Allergy and Clinical Immunology has recently developed guidelines for the prevention, diagnosis and management of food allergy and the management of anaphylaxis. In order to inform dissemination, adaptation and implementation plans, we sought to identify countries that have/do not have national guidelines for food allergy and anaphylaxis.

**Methods:**

Two reviewers independently searched PubMed to identify countries with guidelines for food allergy and/or anaphylaxis from the inception of this database to December 2016. This was supplemented with a search of the Agency for Healthcare Research and Quality’s National Guideline Clearinghouse in order to identify any additional guidelines that may not have been reported in the peer-reviewed literature. Data were descriptively and narratively synthesized.

**Results:**

Overall, 5/193 (3%) of countries had at least one guideline for food allergy or anaphylaxis. We found that one (1%) country had a national guideline for the prevention of food allergy, three (2%) countries had a guideline for the diagnosis of food allergy and three (2%) countries had a guideline for the management of food allergy. Three (2%) countries had an anaphylaxis guideline.

**Conclusions:**

This study concludes that the overwhelming majority of countries do not have any national clinical practice guidelines for food allergy or anaphylaxis.

## Background

The increase in the prevalence of allergic diseases seen over recent decades has resulted in food allergy and anaphylaxis emerging as important clinical conditions globally [1–4]. Concerns have been expressed about the clinical care patients with these conditions receive [5–7]. In an attempt to improve clinical care and outcomes, a number of professional and national organizations have developed clinical practice guidelines for a range of communicable and non-communicable disorders. Much of this activity has however been undertaken in high-income country settings, this reflecting the relative lack of infrastructure, capacity and financial resources available in many low- and middle-income countries (LMICs) to develop their own national guidelines. One way of bridging this gulf is to adapt and customize existing international guidelines for use in countries that do not have their own clinical guidelines [8].

The European Academy of Allergy and Clinical Immunology (EAACI) has recently produced international clinical practice guidelines on the primary prevention [9], diagnosis and management of food allergy [10] and the management of anaphylaxis [11]. In order to inform deliberations on the global dissemination, adaptation and implementation of these EAACI guidelines, we sought to identify which countries had and did not have their own clinical guidelines for food allergy and anaphylaxis.

## Methods

### Search strategy

We searched PubMed database using the search terms “(guideline OR practice parameter) AND (food allergy OR anaphylaxis)”. In addition, we searched the Agency for Healthcare Research and Quality’s (AHRQ) National Guideline Clearinghouse to identify any guidelines that may not have been reported in the peer-reviewed literature [12]. There was no time limit on the searches. Our searches were originally undertaken in June 2016 and were then refreshed in December 2016.

### Guideline selection

Two reviewers independently screened the search results to identify documents that were formally labelled as national guidelines. From these, we selected publications that were directed towards healthcare professionals, had a clear methods section and assigned strength of evidence to recommendations. Duplicate entries were removed and, in cases where there were updates of national guidelines, we selected the most comprehensive and/or most recent version of the guideline. Discrepancies were resolved through discussion or arbitration by a third person if agreement could not be reached.

### Data extraction

We independently extracted data on the body that produced these guidelines, the year of publication and the domain(s) of interest in relation to food allergy and/or anaphylaxis. Discrepancies were resolved through discussion or independent arbitration, if necessary.

### Data synthesis

We undertook a descriptive analysis to identify the number and percentage of countries that had guidelines for food allergy and/or anaphylaxis. The denominator for the number of countries in the world (n = 193) was taken from the list of the United Nations (UN) Member States [13]. We then divided the food allergy guidelines into sub-domains focusing on aspects of prevention, diagnosis and management.

## Results

Our searches of PubMed identified a total of 855 hits from which we selected 60 papers for detailed analysis. Of these, seven satisfied our inclusion criteria. Searching the AHRQ website failed to identify any additional guidelines. We in addition found 11 pan-national guidelines for food allergy prevention (n = 5) [9, 14–17], diagnosis (n = 2) [10, 14] and management (n = 3) [10, 14, 18], and anaphylaxis (n = 3) [11, 19, 20]. “Appendix” details documents that appeared not to meet our inclusion criteria, but which we were unable to fully assess.

### Overall findings

We found that 5/193 (3%) countries had at least one guideline on food allergy or anaphylaxis (Table 1).

**Table 1:** Countries with one or more clinical guidelines for food allergy and/or anaphylaxis

Countries	Food allergy			Anaphylaxis (Y/N)
	Prevention (Y/N)	Diagnosis (Y/N)	Management (Y/N)	
Germany	N	Y	Y	N
Singapore	N	N	Y	N
Spain	N	N	N	Y
UK	N	Y	N	Y
USA	Y	Y	Y	Y
Total	1	3	3	3

### Food allergy

Four (2%) countries had guidelines on some aspect of food allergy (see Table 1). These comprised of one (1%) covering aspects of food allergy prevention, three (2%) dealing with diagnosis, and three (2%) dealing with food allergy management (Table 2).

**Table 2:** National guidelines for food allergy

Country	Source	Published	URL
*Prevention*			
USA	American Academy of Allergy, Asthma and Immunology (AAAAI); the American College of Allergy, Asthma and Immunology (ACAAI); and the Joint Council of Allergy, Asthma and Immunology (JCAAI)	2014	https://www.aaaai.org/Aaaai/media/MediaLibrary/PDF%20Documents/Practice%20and%20Parameters/Food-Allergy-A-Practice-Parameter-Update-2014.pdf
*Diagnosis*			
Germany	German Society of Allergology and Clinical Immunology (DGAKI), the Physicians’ Association of German Allergologists (ADA) and the Society of Pediatric Allergology (GPA) together with the Swiss Society of Allergology. German Society for Allergology and Clinical Immunology	2011	http://onlinelibrary.wiley.com/doi/10.1111/j.1610-0387.2008.06889.x/abstract https://www.thieme-connect.com/DOI/DOI?10.1055/s-0030-1256476
UK	National Institute for Health and Care Excellence (NICE)	2016	https://www.nice.org.uk/guidance/cg116/evidence/full-guideline-136470061
USA	National Institute of Allergy and Infectious Diseases American Academy of Allergy, Asthma and Immunology (AAAAI); the American College of Allergy, Asthma and Immunology (ACAAI); and the Joint Council of Allergy, Asthma and Immunology (JCAAI)	2010, 2014	http://www.jacionline.org/article/S0091-6749%2810%2901566-6/fulltext https://www.aaaai.org/Aaaai/media/MediaLibrary/PDF%20Documents/Practice%20and%20Parameters/Food-Allergy-A-Practice-Parameter-Update-2014.pdf
*Management*			
Germany	German Society of Allergology and Clinical Immunology (DGAKI); Medical Association of German Allergologists (ADA); German Society of Pediatric Allergology	2009	http://www.ncbi.nlm.nih.gov/pubmed/19371249
Singapore	Academy of Medicine, Singapore (AMS) and the Ministry of Health	2010	http://smj.sma.org.sg/5107/5107cpg1.pdf
USA	National Institute of Allergy and Infectious Diseases American Academy of Allergy, Asthma and Immunology (AAAAI); the American College of Allergy, Asthma and Immunology (ACAAI); and the Joint Council of Allergy, Asthma and Immunology (JCAAI)	2010	http://www.jacionline.org/article/S0091-6749%2810%2901566-6/fulltext https://www.aaaai.org/Aaaai/media/MediaLibrary/PDF%20Documents/Practice%20and%20Parameters/Food-Allergy-A-Practice-Parameter-Update-2014.pdf

### Anaphylaxis

Three (2%) of countries has guidelines dealing with anaphylaxis (see Table 3).

**Table 3:** National guidelines for the management of anaphylaxis

Country	Source	Published	URL
Spain	Sociedad Espanola de Alergologia e Inmunologia Clinica	2011	https://www.ncbi.nlm.nih.gov/pubmed/?term=cardona+v+and+guideline+anaphylaxis+2011
UK	National Institute for Health and Care Excellence	2011	https://www.nice.org.uk/guidance/cg134
US	Joint task force on practice parameters, representing the American Academy of Allergy, Asthma and Immunology (AAAAI); the American College of Allergy, Asthma and Immunology (ACAAI); and the Joint Council of Allergy, Asthma and Immunology	2015	https://www.ncbi.nlm.nih.gov/pubmed/?term=Anaphylaxisda+practice+parameter+update+2015

## Discussion

### Summary of principal findings

This analysis of the international literature has found that only a minority of countries have any formally produced clinical practice guidelines for food allergy and anaphylaxis with the major gaps being in African and Asian countries. This is a concern considering that food allergy and anaphylaxis now affect people globally and furthermore they are associated with significant morbidity and, in some cases, mortality [21, 22].

### Strengths and limitations

The key strengths of this study are that we formally searched the principal biomedical database, namely PubMed using established systematic search techniques and that these searches were from the inception of this database and were not restricted by language. Furthermore, we cross-checked the results with AHRQ’s National Guideline Clearinghouse, which is the foremost repository of clinical guidelines.

The main limitation is that we may have missed some guidelines that were either not formally published in the peer-reviewed literature and/or were not available in English. We may also have missed guidelines that are in development.

### Interpretation in the light of previous published research

This is, as far as we are aware, the first truly international overview of guidelines available for both food allergy and anaphylaxis. It builds on a recent comparison of food allergy guidelines [23] and our earlier study focused on anaphylaxis, both of which found a more limited number of guidelines and considerable variation in the recommendations contained in these guidelines [24]. This work subsequently contributed to the creation of an International Consensus statement on anaphylaxis [25].

## Conclusions

The findings of this study point to considerable gaps in the availability of national guidelines for food allergy and anaphylaxis. EAACI and other international guideline bodies should consider working with the countries that currently have no national guidelines to produce clinical practice guidelines on food allergy and anaphylaxis that are language specific and tailored to their local needs. Furthermore we suggest compiling an online database on the EAACI website which logs adapted versions of the guideline and also proactively sending details of these adapted guidelines for listing on the AHRQ’s National Guideline Clearinghouse: https://www.guideline.gov/.

## Appendix

See Table 4.

**Table 4:** National guidelines that we were unable to formally assess

Name	Country	Year	Reason for exclusion
Practice guidelines 2005: management of anaphylaxis	Japan	2006	In Japanese
Allergies in the 1st life year. The allergy prevention guideline 2004 of the allergy prevention action alliance (abap)	German	2005	In German
Anaphylaxis guideline—its incentives and pre-hospital care	Japan	2016	In Japanese
Japanese guideline for food allergy 2012 specific type of food allergy	Japan	2012	In Japanese
Japanese pediatric guideline for food allergy 2012]	Japan	2012	In Japanese
JSA anaphylaxis guideline—importance of basic management and prevention	Japan	2015	In Japanese
Practice guidelines for food allergy	Japan	2012	In Japanese
New approach and recommendation in JAGL2007 (Japanese guideline for the diagnosis and treatment of allergic diseases 2007)	Japan	2009	In Japanese
Evidence based recommendations for the diagnosis and management of cow’s milk allergy in Chinese infants	China	2013	In Chinese
